# Intersectional challenges facing women in radiology and oncology

**DOI:** 10.1093/bjr/tqag038

**Published:** 2026-03-09

**Authors:** Zsuzsanna Iyizoba-Ebozue, Evelyn Carroll, Jade Scott-Blagrove, Chitra Viswanathan, Anu E Obaro

**Affiliations:** Department of Clinical Oncology, Leeds Cancer Centre, Leeds, United Kingdom; Leeds Institute of Medical Research, University of Leeds, Leeds, United Kingdom; Department of Radiology, Mayo Clinic, Rochester, MN, United States; Cambridge University Hospitals NHS Foundation Trust, United Kingdom; University of Texas MD Anderson Cancer Center, Houston, TX, United States; King’s College Hospital NHS Foundation Trust, London, United Kingdom

**Keywords:** intersectionality, equity, radiology, oncology, women, racial disparity, disability, work-life balance, sexual orientation

## Abstract

As a group of diverse co-authors working in the fields of clinical radiology and oncology, we have first-hand experience of the systems that impact medical careers. Women remain underrepresented in most spaces and often face a complex set of obstacles exacerbated by additional protected characteristics such as race, disability and sexual orientation. The impact of flexible working and care giving responsibilities on career development as well as leadership opportunities further widens the gap between under- and overrepresented groups. In this commentary we describe the intersectional challenges faced by women in our specialties and the challenge of navigating these spaces to achieve career success. We explore the various forms of discrimination and its impact on representation in medicine. Potential strategies and solutions aimed at promoting a more equitable specialty are also highlighted.

## Introduction: medicine mirrors societal inequity

Across society, women and people from marginalised groups remain significantly underrepresented in senior leadership roles. The 2024 Gender Equality Report & Ranking found that women represented only 22% of executives and 27% of senior managers among 4000 global companies.^1^ These disparities reflect longstanding societal inequities, including biased promotion practices, limited access to sponsorship, and exclusion from influential networks.

Medicine mirrors these patterns; although diversity at entry level has increased, current structures continue to replicate the same patterns seen in corporate environments. Within the National Health Service (NHS) women make up 77% of the workforce, but across 213 Trusts, women’s representation on trust boards is only 44.7%, albeit a 5% improvement since 2017 (range 15.4% to 77.8%).^2^

Despite apparent progress, the medical profession remains characterised by longstanding traditions and hierarchical structures which are challenging to navigate. Systemic barriers persist within the field, and these obstacles are often disproportionately amplified for certain groups. Intersectionality, first described by Kimberlé Crenshaw, highlights how systems of oppression such as racism, sexism, classism, ableism, or discrimination based on sexual orientation, overlap creating unique experiences for marginalised groups.^3^ It highlights the interconnected nature of social categorisations and their compounding effects on individuals, particularly those who occupy multiple marginalised identities.

Although recent rhetoric opposes the value of diversity and inclusion, the case for tackling inequity in medicine and promoting inclusivity has been well made.^4^^,^^5^ Women have steadily increased their presence in radiology and oncology and according to data from the Royal College of Radiologists (RCR) in the UK, account for 37% and 50% respectively.^6^^,^^7^ However, applying an intersectional lens to our specialty reveals persistent inequalities faced by women, shaped by overlapping challenges of gender, race, disability, sexual orientation, care-giving responsibilities and the pressures of research demands. In this commentary, we consider the experiences of women as the common thread and explore other protected characteristics; acknowledging that this is not exhaustive discussion but drawing on issues of which we have lived experience.

## Women in academia

### Current challenges

Historically, medical academia has evolved around Eurocentric and patriarchal norms, which has frequently marginalised the contributions of women. Women, in general, are under-represented in first and last authorship,^8^ a proxy metric for academic impact and success. Issues including systemic biases and cultural invisibility, shape (and impede) pathways to academic leadership in distinct ways, such as limited mentorship opportunities, unequal evaluation criteria, and rigid working patterns. Even if leadership positions are achieved, many women remain on the periphery of networks, with lack of recognition of their contributions and are frequently overlooked by decision-making bodies.^9^

### Best practices

Gender-responsive leadership training including programmes within the NHS Leadership Academy have improved retention and progression of women into decision-making roles. Adopting a structured toolkit such as the Women-in-Radiology group designed by the American Association for Women in Radiology provides a support system with benefits to the individual and institution, promoting the advancement of female colleagues.^10^

## Women of colour

### Current challenges

Recognising that summarising ethnically diverse groups into a broad category as inherently problematic, the experience of Women of Colour, particularly Black women, illustrates the intersectional challenge of gendered and racialised inequities. Although there are over 25, 0000 professors across disciplines in the UK, 8150 of whom are women, only 70 are Black; less than 0.5% - the smallest proportion of the UK Professoriate.^11^ Conversely, there are 560 Asian female and 6,660 White female professors.^11^ Among the Black female professors, only six are of medicine and at the time of writing there is only one Black, female professor of radiology in the UK.^12^ Professorships confer authority over funding, research topics and training pathways, and the near absence of Black women in these roles demonstrates long-standing exclusion from this decision making space. Furthermore, ‘cultural taxation’ burdens Women of Colour, exploiting their unique perspectives for diversity work in frequently unpaid and undervalued efforts to create inclusive spaces without addressing the structural inequities which limit their authority.^13^^,^^14^

### Best practices

In the UK, targeted leadership development programmes such as 100 Black Women Professors Now^15^ and Generation Delta^16^ support Women of Colour to progress into senior academic roles through mentoring, strategic advice and institutional accountability. In the US, the American Association of Medical Colleges’ Advancing Gender Equity in Academic Medicine provides an extensive evidence-based resource of dedicated programmes and initiatives to increase promotion and retention of racialised women.^17^

## Women with disabilities

### Current challenges

Disability is often viewed through a negative lens in the medical community, with the assumption that it diminishes clinical capability. This ableist perspective is compounded by structural ableism—inaccessible environments with lack of reasonable adjustments and inflexible policies which further marginalize those with disabilities.^18^ Doctors with disabilities are more likely to experience discrimination, pay inequities and limited career advancement which contributes to underreporting and concealment of disabilities.^19^ When intersected with other identities such as being a woman, transgender or nonbinary, or of a different race these challenges multiply and lead to a diminished sense of belonging and increased burnout.^20^^,^^21^

Disability alone does not account for the challenges faced by medical professionals with disabilities; overlapping systems of oppression exacerbate their discrimination. Addressing ableism requires recognising how race, gender, and other factors intersect to shape their experiences.

### Best practices

The Docs with Disabilities Initiative drives policy change to improve recruitment, retention and wellbeing for disabled clinicians.^22^ Disability-inclusive leadership training and collective accountability have helped to increase representation.

## LGBTQ+ women

### Current challenges

LGBTQ+ women face unique challenges stemming from their intersectional identities and often feel pressured to conceal their identity to conform to heteronormative expectations of healthcare professionalism. Lack of female LGBTQ+ representation in radiology and oncology fosters isolation and invisibility, while microaggressions, distasteful comments and exclusion from mentorship opportunities are common. The inability to be authentic at work leads to emotional distress, lower job satisfaction, and potential mental health decline.^23^ Absence of role models or mentors with a shared identity means LGBTQ+ women often face challenges in navigating professional environments that may not recognise or validate their experience, creating distinctive vulnerabilities that can impact professional development and exacerbate workplace inequities.

### Best practices

Rainbow Badge Initiatives like those supported by the NHS and active promotion of LGBT+ inclusion e.g. by the Radiological Society of North America (RSNA) increases staff confidence in being open about their identity and strengthens institutional accountability.^24^^,^^25^

## Women with caregiving responsibilities

### Current challenges

Balancing the demands of clinical roles with caregiving responsibilities remains a significant barrier for women. Although flexible work arrangements are becoming increasingly common (39% of radiology and 42% of oncology consultants in the UK work less than full-time (LTFT)),^6^^,^^7^ they are predominately used by women. This often leads to exclusion from leadership tracks with reduced career advancement opportunities for women and direct impact on pay.^26^

### Best practices

The NHS offers several return-to-work programmes such as the Return to Work Mentoring programme and Supported Return to Training (SuppoRTT) which provide training updates to skills and support for clinicians transitioning back to work following a period of absence.^27^^,^^28^ Dedicated LTFT training support from the Royal College of Radiologists (RCR), along with LTFT local champions and guidance from the British Medical Association have expanded LTFT rights and access to flexible working.^29^^,^^30^ Structured return to work and family-friendly policies are shown to provide smoother transitions back to work and support part-time career progression.^26^

## Future state: building an equitable and inclusive specialty

The work of creating truly equitable spaces in radiology and oncology must understand intersectionality at its core. Recognising that the more protected characteristics a person has, the more barriers they are likely to face, will better inform systemic solutions for career retention and progression.

Achievable goals for radiology and oncology would include:

- transparent, audited recruitment and promotion processes
- institutional mentorship and sponsorship programmes
- standardised accessibility standards
- normalised flexible work and job-sharing leadership roles
- protected time for research and leadership development
- equity-centred leadership training
- robust organisational accountability structures

Some of these can be developed at the specialty level (i.e through Royal Colleges or Societies), while others require local, organisational and individual level input.

On an individual basis, those of us in leadership positions can proactively offer opportunities and advocacy to women from underrepresented groups. Targeted formal and informal mentorship builds authentic communities of support that are diverse in their nature.

Institutions can amplify the voices of women from diverse backgrounds, recognising that their experiences offer invaluable insights into creating an inclusive profession and culturally competent patient care.

Much of the challenge of intersectional equity is due to the perceived archetype of the doctor and long-standing unchallenged structures. Therefore, rather than merely navigating the complexities of our identities, we must redefine professional environments to encourage, value and acknowledge contributions of women.
